# Adipokines, insulin resistance, and adiposity as a predictors of metabolic syndrome in child survivors of lymphoma and acute lymphoblastic leukemia of a developing country

**DOI:** 10.1186/s12885-017-3097-8

**Published:** 2017-02-13

**Authors:** Lourdes Barbosa-Cortés, Mardia López-Alarcón, Juan Manuel Mejía-Aranguré, Miguel Klünder-Klünder, María del Carmen Rodríguez-Zepeda, Hugo Rivera-Márquez, Alan de la Vega-Martínez, Jorge Martin-Trejo, Juan Shum-Luis, Karina Solis-Labastida, Enrique López-Aguilar, Guadalupe Matute-González, Roberto Bernaldez-Rios

**Affiliations:** 1 Medical Nutrition Research Unit, 21st Century Pediatric Hospital, National Medical Center, MexicanInstitute of Social Security, México City, México; 2 Health Research Coordination, 21st Century Pediatric Hospital, National Medical Center, Mexican Institute of Social Security, México City, México; 3 Department of Community Health Research, Federico Gómez Children’s, México Secretary of Health, and Research Committee, Latin American Society for Pediatric Gastroenterology, Hepatology and Nutrition (LASPGHAN), México City, México; 4 Department of Hematology, Pediatric Hospital, 21st Century National Medical Center, Mexican Institute of Social Security, México City, México; 5 Department of Hemato-Oncology, General Hospital of México, México Secretary of Health, México City, México; 6 Department of Oncology, Pediatric Hospital, 21st Century National Medical Center, Mexican Institute of Social Security, México City, México; 7 Medical Nutrition Research Unit, 21st Century Pediatric Hospital, National Medical Center, Mexican Institute of Social Security, Avenida Cuauhtémoc 330 Col. Doctores, México City, C.P. 06720 México

**Keywords:** Childhood cancer survivors, Acute lymphoblastic leukemia and lymphoma, Metabolic syndrome, Adipokines, Insulin resistance, Obesity

## Abstract

**Background:**

There is a growing body of evidence indicating that pediatric survivors of cancer are at a greater risk of developing metabolic syndrome. This study evaluated some probable predictors of metabolic syndrome (MS), such as leptin and adiponectin concentrations, the leptin/adiponectin ratio, insulin resistance, and adiposity, in a sample of child survivors of lymphoma and leukemia in Mexico City.

**Methods:**

Fifty two children (leukemia *n* = 26, lymphoma *n* = 26), who were within the first 5 years after cessation of therapy, were considered as eligible to participate in the study. Testing included fasting insulin, glucose, adipokines and lipids; body fat mass was measured by DXA. The MS components were analyzed according to tertiles of adipokines, insulin resistance, and adiposity. Comparisons between continuous variables were performed according to the data distribution. The MS components were analyzed according to tertiles of adipokines, insulin resistance, and adiposity. With the purpose of assessing the risk of a present MS diagnosis, odds ratios (OR) with a 95% confidence interval (95% IC) were obtained using logistic regression analysis according to the various metabolic markers.

**Results:**

The median children age was 12.1 years, and the interval time from the completion of therapy to study enrollment was 4 years. Among the MS components, the prevalence of HDL-C low was most common (42%), followed by central obesity (29%). The HOMA-IR (OR 9.0, 95% CI 2.0; 41.1), body fat (OR 5.5, 95% CI 1.6; 19.3), leptin level (OR 5.7, 95% CI 1.6; 20.2) and leptin/adiponectin ratio (OR 9.4, 95% CI 2.0; 49.8) in the highest tertile, were predictive factors of developing MS; whereas the lowest tertile of adiponectin was associated with a protective effect but not significant.

**Conclusions:**

Biomarkers such as HOMA-IR, leptin and leptin/adiponectin are associated with each of the components of the MS and with a heightened risk of suffering MS among children survivors of cancer. Given the close relationship between MS with risk of developing type 2 diabetes and cardiovascular disease, it is imperative to implement prevention measures in this population and especially in developing countries where these pathologies have become the leading cause of death.

## Background

The increase in the survival rates of child cancer patients is accompanied by an increase in the likelihood to develop long-term adverse effects. Specifically, survivors of ALL (Acute lymphoblastic leukemia) and lymphoma are at risk of developing metabolic abnormalities, such as obesity, impaired glucose tolerance, insulin resistance, metabolic syndrome, cardiovascular disease, and diabetes [1–4].

On the other hand, the excess adipose tissue that accompanies obesity alters the release of adipokines, including leptin and adiponectin, which are known to affect insulin sensitivity and vascular functionality, thereby, increasing the risk of MS [5]. In fact, low adiponectin and high leptin concentrations are observed in children with MS and insulin resistance [5, 6]. This imbalance between pro and anti-inflammatory adipokines elicits low degree systemic inflammation, mainly associated with insulin resistance [7, 8]. Even more, both, hypoadiponectinemia and hyperleptinemia are considered to be independent risk factors of the development of type 2 diabetes and cardiovascular diseases [5, 7, 9–13]. Owing to this fact, its plasmatic concentrations has been proposed as a candidate biomarkers to identify metabolic alterations including those of MS [14]. However, among children survivors of cancer it is not exactly known how and to what extent IR, adiponectin and leptin are associated with each of the components of MS.

Therefore the aim of this study was to evaluate some probable predictors of MS, such as leptin and adiponectin concentrations, the leptin/adiponectin ratio, insulin resistance, and adiposity, in a sample of child survivorsof ALL and lymphoma. To exclude random effects, these factors were re-analyzed after 6 months.

## Methods

### Subjects

Children referred to the Pediatric Hospital from the Mexican Institute of Social Security (IMSS) in Mexico City who previously diagnosed with ALL or lymphoma, and who were within the first 5 years after cessation of therapy, without relapse, no second neoplasm or bone marrow transplants were considered as eligible to participate in the study.

From 61 potential candidates, 52 children met the selection criteria (39 boys and 13 girls); Children who accepted to participate were asked to attend the pediatric hospital on two occasions (baseline examination and 6 months later). This study was approved by the Research and Ethics Committee of the Pediatric Hospital at the Mexican Social Security Institute (2005/3603/071). We obtained written informed consent from parents and informed assent from children.

### Procedures

#### Anthropometry

Body weight was measured with an electronic scale (TANITA BWB-700, Tanita Corporation., Tokyo, Japan) with the subjects wearing light-weight clothing. Height was measured to the nearest 0.1 cm with a wall-mounted stadiometer (SECA 222, SECA Corp., Oakland Center, Columbia, MD, USA). Waist circumference was measured at the level of the superior iliac crest to the nearest 0.1 cm, and the values were changed to percentiles according to Fernandez [15]. Body mass index (BMI) and BMI percentiles for age and sex were calculated according to the Center of Disease Control (CDC) normative curves using the computer software Epi-Info (Obesity was defined conventionally as ≥95^th^ percentile, overweight as 85^th^ to 94^th^ percentile, and underweight as <5^th^ percentile [16]. Pubertal stage was determined on the basis of the children’s self-assessment by using Tanner’s standard photographs for pubic hair and genital development. We did not measure testicular size in the children [17].

#### Body composition

Body fat percentage (BFP) was determined by dual-energy X-ray absorptiometry (DXA) using a GE Lunar Prodigy Advance scanner (software version 9.0; GE Medical Systems, Madison, WI, USA). The machine was operated by only one technician.

#### Metabolic syndrome definition (MS)

Metabolic syndrome was defined in accordance with the guidelines of the International Diabetes Federation (IDF) [18], with the exception of the cutoff point for blood pressure, which was obtained from the National High Blood Pressure Education Program Working Group on high blood pressure in children and adolescents [19]. The rationale is that the cutoff point for blood pressure used by the IDF does not properly represent the effects of age, sex, and stature of children and adolescents. If the IDF criterion is used for blood pressure, the prevalence of high blood pressure would be underestimated because very few children exceed these values. Therefore, children with metabolic syndrome were those with central obesity based on a waist circumference (WC) within ≥90^th^ percentile adjusted for age, sex, and Hispanic ethnicity and the presence of two or more of the following features: a) hypertension as diastolic or systolic blood pressure within ≥90^th^ percentile for age and sex; b) elevated triglyceride levels of ≥150 mg/dL; c) low high-density lipoprotein cholesterol (HDL-c) level of <40 mg/dL; and d) fasting glucose level of ≥100 mg/L.

#### Analytical methods

Venous blood samples were collected between 8:00 and 9:00 AM after a 12-h overnight fast. Serum aliquots were separated and frozen at −80 °C until biochemical determination. Fasting glucose concentrations were determined by the glucose oxidase method (Beckman Coulter Glucose Analyzer; Beckman Coulter, Brea, CA, USA). Triglycerides, total cholesterol, and HDL-C were measured by the enzymatic colorimetric method (SPIN 120 automatic analyzer Shenzhen, Mindray) with commercially available kits. Leptin and adiponectin levels, vascular cell adhesion molecule (vCAM) and intercellular cell adhesion molecule (iCAM) were determined in duplicate by ELISA (Human Adiponectin, Leptin, vCAM and iCAM ELISA Kits, Millipore, St. Charles, MO, USA), and insulin levels were determined by radioimmunoassay using a commercial kit (Linco Research, St. Charles, MO, USA). Insulin resistance (IR) was determined based on the fasting insulin and glucose concentrations using the Homeostatic Model Assessment of Insulin Resistance [HOMA-IR = (fasting insulin (μU/ml) * fasting glucose (mmol/L)/22.5) [20]. Insulin resistance was defined according to the percentile distribution by age and sex proposed for Mexican children and adolescents [21].

#### Statistical analysis

All statistical analyses were performed using SPSS v.18.0 (SPSS Inc., Chicago, IL, USA) and STATA/SE v.11.0 (STATA Corp., College Station, TX, USA). The frequency of MS and each component of MS were obtained, and differences in proportions were assessed by Fisher’s exact test. The data were grouped according to the following characteristics: with or without MS features, with or without MS diagnoses, by different cancer treatments, as well as the nutritional status. Anthropometric, metabolic, and inflammatory markers data were tested for normal distribution based in skewness and kurtosis and Shapiro-Wilk test; however, as log transformation did not achieve normal distribution of some of the studied variables such as adiponectin, we show the medians instead of the log transformations of the non-normally distributes variables. Comparisons between continuous variables were performed with Student’s *t* test or the Mann–Whitney *U* test according to data distribution. The MS components were analyzed according to tertiles of adipokines, insulin resistance, and adiposity. Differences in metabolic features between the aforementioned tertile categories were obtained using robust linear regression analysis. With the purpose of assessing the risk of a present MS diagnosis, odds ratios (OR) with a 95% confidence interval (95% IC) were obtained using logistic regression analysis according to the various metabolic marker tertiles; these data were adjusted by sex, age, treatment, and diagnosis (lymphoma or ALL). The associations were re-evaluated at a 6-month follow-up to validate that the findings observed were not due to chance. Additionally, an analysis between the metabolic variables with the changes observed between baseline and 6-month measurements was performed to identify which variables were most influenced by long-term changes. The changes between first evaluation and 6-month measurements were analyzed in tertiles using the middle tertile as a reference value because it was assumed that this tertile did not show changes between the baseline and 6-month measurement. Differences were considered significant at *p* < 0.05 with all *P* values based on two-sided tests.

## Results

Demographic and clinical characteristics of participant children are shown in Table 1. At entrance to the study, the median age was 12.1 years, and the interval time from the completion of cancer therapy to study enrollment was 4 years. At enrollment, 38.4% of children were classified as overweight or obese; after 6 months, the incidence of overweight and obesity increased to 44%. In the same table, the child survivors of leukemia and lymphoma were classified according the presence of MS diagnosis at first and 6 months assessments, and as expected, the insulin levels, HOMA-IR index, leptin levels (ng/mL), leptin/adiponectin ratio, and BFP, differed significantly between children with and without MS.

**Table 1:** Metabolic characteristics of child survivors of acute lymphoblastic leukemia and lymphoma according to MS

Variables	Firts Assessesment						After 6 months			
	All		No MS		MS		No MS		MS	
*n*	52		45		7		42		10	
Sex (M) *n* (%)	39	75	33	73	6	86	31	74	8	80
Current age (Y)^a^	12.1	(7.1, 17.0)	12.2	(7.1, 17.0)	11.1	(7.1, 17.1)	12.1	(7.1, 17.0)	11.6	(7.1, 17.1)
Age at cancer diagnosis (Y)^a^	5.3	(1.0, 13)	6	(0.8, 13.0)	4	(2, 11.1)	6.1	(2.1, 13.0)	4.1	(0.08, 12.1)
Time since completion of therapy (Y)^a^	4	(2, 5)	4	(2, 5)	4	(2, 5)	4	(2, 5)	4	(2, 5)
Diagnosis (*n* %)										
ALL	26	50	22	48.9	4	57.1	20	47.6	6	60.0
Lymphoma	26	50	23	51.1	3	42.9	22	52.4	4	40.0
Pubertal stage (*n* %)										
1	33	63.5	28	62.2	5	71.4	18	42.9	7	70.0
≥ 2	19	36.5	17	37.8	2	28.6	24	57.1	3	30.0
Treatment recived (*n* %)										0.0
Ch	35	67.3	31	68.9	4	57.1	28	66.7	7	70.0
Ch + R	17	32.7	14	31.1	3	42.9	14	33.3	3	30.0
Nutritional Status (*n* %)										
Normal	29	55.8	28	62.2	1	14.3	27	64.3	0	0
Overweight/Obesity	20	38.4	14	31.1	6	85.7	13	31.0	10	100
Low weight	3	5.8	3	6.7	0	0	2	4.8	0	0
MS Components median (min, max)										
WC (cm)	70.5 (51.1, 105.7)		69.9 (51.1, 94.8)		78.1 (60.4, 105.7)*		50 (10, 100)		90 (90, 100)*	
SBP (pc)	47 (2, 99)		42.5 (2, 93)		58 (14, 99)		16 (1, 70)		65 (19, 95)*	
DBP (pc)	76 (15, 99)		75 (15, 97)		94 (19, 99)*		54 (11, 92)		94 (66, 99)*	
Triglicerydes (mg/dL)	96.6 (34, 231)		85 (34, 205)		182 (117, 231)*		80 (28, 259)		169 (104, 264)*	
HDL-C (mg/dL)	40 (18, 84)		40 (18, 84)		40 (18,46)		45 (20, 80)		37 (22, 68)	
Glucose (mg/dL)	89 (74, 116)		89 (74, 116)		93 (76, 112)		93 (77, 105)		90 (83, 115)	
Others metabolic variables median (min, max)										
Insulin (μU/mL)	18.9 (4.3, 55)		18.7 (4.3, 55)		28.2 (17.5, 50)*		22.8 (3.4, 56.6)		31.5 (24.5, 64)*	
HOMA-IR	4.3 (0.79, 13.9)		4.2 (0.79, 12.7)		7.4 (3.3, 13.9)*		5.2 (0.68, 13.8)		7.3 (5.2, 15.4)*	
Adiponectin (μg/mL)	20.3 (1.3, 70)		23.4 (1.3, 70)		17.5 (11.7, 25.4)		20.2 (0.64, 57.3)		20.3 (8.3, 37.2)	
Adiponectin per Kg fat (μg/mL/kg)	1.4 (0.07, 8.0)		1.6 (0.07, 8.0)		1.0 (0.38, 3.9)*		1.7 (0.06, 14.3)		0.90 (0.27, 3.8)	
Leptin (ng/mL)	10.1 (1.2, 30.9)		8.0 (1.2, 30.9)		17.5 (8.5, 28.6)*		6.5, (1.1, 30.9)		22 (9.4, 28.2)*	
Leptin (μg/mL/kg)	0.69 (0.11, 3.9)		0.69 (0.11, 3.9)		1.0 (0.35, 3.7)		0.65 (0.11, 4.8)		1.0 (0.30, 4.2)	
Leptin to Adiponectin ratio	0.44 (0.06, 11)		0.35 (0.06, 11)		0.98 (0.69, 1.5)*		0.34 (0.05, 10.4)		1.1 (0.31, 1.7)*	
Body Fat (%)	29.9 (8.3, 46.7)		27.6 (8.3, 44.4)		38.3 (31.2, 46.7)*		25.5 (7.2, 45.5)		39.8 (29.6, 49.3)*	
VCAM (ng/mL)	925 (425, 1730)		930 (425, 1730)		769 (490, 1697)		753 (377, 2045)		683.9 (514, 1081)	
ICAM (ng/mL)	459 (254, 1079)		468 (254, 1079)		412 (325, 685)		456.5 (184, 137)		404 (199, 631)	

^a^Values are median (min, max); *Abbreviations*: *MS* Metabolic syndrome, *Ch* chemotherapy, *Ch + R* chemotheraphy + radiotherapy, *HOMA-IR* Homeostatic Model Assessment of Insulin Resistance, *VCAM* Vascular cell adhesion molecule, *ICAM* Intercellular adhesion molecule; Comparations were assessed using Mann–Whitney *U*-test due non-normal distribution of the data, and categorized data were analyzed using Fisher’s Test; * *p* value < 0.05

Among the MS components, the prevalence of HDL-C low was most common (42.3%), followed by central obesity (28.8%). At 6 months, the number of patients with a diagnosis of MS increased to 19%. Additionally, increasing trends in the number of children with alterations in triglycerides (17.3 to 23.1%), and glucose levels (7.7 to 17.3%) and in the number who presented with 4 criteria for the diagnosis of MS (2 to 7.7%) were observed (Table 2).

**Table 2:** Prevalence and components of metabolic syndrome in child survivors of acute lymphoblastic leukemia and lymphoma

MS	First assessment	After 6 months
*n*	52	52
WC^a^ ≥90 pc^b^	15 (28.8)	15 (28.8)
Triglycerides ≥150 mg/dL	9 (17.3)	12 (23.1)
HDL-C^c^ <40 mg/dL	22 (42.3)	22 (42.3)
High BP^d^	14 (26.9)	8 (15.4)
Glucose >100 mg/dL	4 (7.7)	9 (17.3)
No. of MS criteria		
0	14 (26.9)	17 (32.7)
1 criteria	19 (36.5)	17 (32.7)
2 criteria	12 (23.1)	8 (15.4)
3 criteria	6 (11.5)	6 (11.5)
4 criteria	1 (1.9)	4 (7.7)
MS	7 (13.5)	10 (19.2)

Values are Number (percentage); *Abbreviations*: *MS* Metabolic syndrome, ^a^ *WC* Waist circumference, *pc* ^b^ Percentile, ^c^ *HDL-C* High density lipoprotein cholesterol, ^d^Systolic blood pressure ≥90 pc or Diastolic blood pressure ≥90 pc

The metabolic characteristics of the child survivors of leukemia and lymphoma according to the treatment received and nutritional status are presented in Tables 3 and 4. The results of the analysis of metabolic markers in all the children who received chemotherapy and those who received radiotherapy besides chemotherapy did not significantly differ. Of all the children, 35 (67%) received chemotherapy only, of whom 15 (47%) were classified as overweight or obese. All of the metabolic syndrome components were similar in the chemotherapy group (normal group vs overweight/obese group), except for waist circumference (*p* < 0.0001) and other metabolic variables such as insulin level (16.3 μU/mL vs 24.9 μU/mL, *p* = 0.010), HOMA-IR index (3.6 vs 5.5, *p =* 0.033), leptin level (4.4 ng/mL vs 18.2 ng/mL, *p* < 0.0001), leptin-to-adiponectin ratio (0.27 vs 0.98, *p* < 0.0001), and fat mass percentage (23.4 vs 38.3, *p* < 0.001). However, the group of children who received chemotherapy plus radiotherapy could not be statistically compared because only five (29%) of the 17 children were classified into the overweight/obese subgroup. However, the overweight/obese children tended to exhibit higher values than the healthy children did, in terms of insulin levels (20.5 μU/mL vs 36.3 μU/mL), HOMA-IR index (4.3 vs 8.3), and leptin level (7.4 pg/mL vs 17.5 pg/mL) (Table 3). The analysis of changes in weight and height between the baseline measurement and 6 months after treatment according to the type of therapy received revealed significant differences in height. The height of the children in the chemotherapy group increased by 2.6 cm (range: 1.7–6.0), while that of the children in the radiotherapy plus chemotherapy group showed a growth of 1.0 cm (range: 0.2–3.5; *p* = 0.035) (Table 4). We wish to indicate that age did not significantly differ between the treatment groups. At 6 months, all of the metabolic syndrome components were similar in the chemotherapy plus radiotherapy group (normal group vs overweight/obese group).

**Table 3:** Metabolic characteristics of child survivors of leukemia and lymphoma according to treatment received at first assessment

	Treatment			
	Chemotherapy *n* = 32^a^		Chemotherapy plus radiotherapy *n* = 17^b^	
Variables	Normal	Overweigth/Obese	Normal	Overweigth/Obese
* n*: number (%)	17	15 (47)	*12*	5 (29)
Sex (M)	12	12	9	4
Current age (Y)	12.1 (8.1, 17.0)	12 (7.1, 17.0)	12.6 (7.1, 16.1)	12.1 (8.0, 17.1)
Age at cancer diagnosis (Y)	6.0 (2.0, 13.0)	4.0 (2.0, 12.1)	6.5 (0.08, 12.1)	7 (1.0, 11.1)
Time since completion of therapy (Y)	4.0 (2.0, 5.1)	4.0 (2.1, 5.1)	3.1 (3.0, 5.0)	4.0 (2.0, 5.0)
Weight (kg)	46.9 (18.1, 91.2)		48.9 (26.2, 102.9)	
Height (cm)	148.4 (116.2, 178)		157.9 (123, 176.1)	
Diagnosis : ALL/Lymphoma	11/6	9/6	4/8	1/4
Pubertal stage				
1	9	13*	5	3
≥ 2	8	2	7	2
MS Components				
WC (pc)	25 (10, 75)	90 (50, 100)*	50 (10, 90)	90 (75, 100)
SBP (pc)	47 (3, 92)	53 (18, 93)	17 (2, 65)	47 (20, 99)
DBP (pc)	76 (15, 94)	82 (57, 99)	75 (19, 96)	89 (39, 99)
Triglicerydes (mg/dL)	76 (34, 149)	114 (50, 231)	72 (35, 164)	188 (148, 210)
HDL-C (mg/dL)	42 (25, 84)	39.5 (26, 78)	42 (18, 64)	43 (18, 48)
Glucose (mg/dL)	90 (75.5, 115.7)	91 (76, 105.7)	86 (74, 112.5)	93 (82, 99)
Others metabolic variables				
Insulin (μU/mL)	16.3 (9.1, 38.7)	24.9 (14.2, 35.3)*	20.5 (4.3, 50)	36.3 (19, 55)
HOMA-IR	3.6 (1.7, 9.5)	5.5 (2.8, 8.2)*	4.3 (0.79, 13.9)	8.3 (4.7, 12.7)
Adiponectin (μg/mL)	23 (1.3, 44.3)	22 (6.8, 70)	19.1 (11.7, 41.2)	16 (1.3, 27.6)
Adiponectin per Kg fat (μg/mL/kg)	1.5 (0.39, 6.7)	1.2 (0.28, 4.9)	1.8 (0.51, 5.1)	0.75 (0.7, 1.8)
Leptin ((μg/mL)	4.4 (2, 13.4)	18.2 (9.8, 30.9)*	7.4 (1.2, 19.0)	17.5 (13.5, 18.5)
Leptin per Kg fat (μg/mL/kg)	0.49 (0.11, 3.0)	1.0 (0.34, 3.7)*	0.46 (0.16, 3.9)	0.72 (0.53, 1.31)
Leptin to Adiponectin ratio	0.27 (0.08, 2.7)	0.98 (0.20, 3.0)*	0.33 (0.06, 0.76)	0.85 (0.67, 11.0)
Body Fat (%)	23.4, (9.9, 36.2)	38.3 (32.0, 46.7)*	27.4 (8.3, 38.1)	37.1 (28.5, 40.2)
VCAM (ng/mL)	1058 (561, 1727)	818 (425, 1730)	856 (586, 1207)	989 (490, 1697)
ICAM (ng/mL)	459 (254, 661)	418 (294, 1079)	500 (265, 952)	398 (324, 598)
MS				
Yes/No	0/17	4/11	1/11	2/3

Values are median (min, max); *Abbreviations*: *MS* Metabolic syndrome, *HOMA-IR* Homeostatic Model Assessment of Insulin Resistance, *VCAM* Vascular cell adhesion molecule, *ICAM* Intercellular adhesion molecule. ^a^Three children of Chemotherapy group were excluded, because they were underweight; Data compared Mann–Whitney *U*-test and categorized data were analyzed using Fisher’s Test, *p value < 0.05; ^b^It was not possible to compare statistically (sample size)

**Table 4:** Metabolic characteristics of child survivors of leukemia and lymphoma according to treatment at 6-months

	Treatment			
	Chemotherapy^a^ *n* = 32		Chemotherapy plus radiotherapy *n* =17	
Variables	Normal	Overweigth/Obese	Normal	Overweigth/Obese
* n*: number (%)	*18*	14 (40)	9	8 (47)
Sex: M	13	11	6	7
Current age (Y)	12.5 (8.0, 17.1)	11.6 (8.0, 17.1)	14.0 (8.4, 17.0)	13.0 (8.1, 18.0)
Age at cancer diagnosis (Y)	6.0 (2, 13)	4.0 (2, 12)	6.0 (0.08, 12)	70 (1.0, 11.1)
Time since completion of therapy (Y)	4.0 (2.1, 5.0)	4.0 (2.1, 5.0)	3.1 (3.0, 5.0)	4.0 (2.0, 5.0)
Δ Body weight (kg) (6 mo - first assessement)	1.6 (−2.5, 5.3)		2.0 (−1.2, 5.1)	
Δ Height (cm) (6 mo - first assessment)	2.6 (1.7, 6.0)		1.0 (0.2, 3.5)*	
Diagnosis : ALL/Lymphoma	11/7	9/5	3/6	2/6
Pubertal stage				
1	8	9	3	2
≥ 2	10	5	6	6
MS Components				
WC (pc)	25 (10, 50)	90 (35, 100)*	30 (10, 50)	83 (50, 90)**
SBP (pc)	15.5 (0, 55)	64 (2, 95)*	13 (1, 38)	21 (3, 65)
DBP (pc)	54 (22, 92)	87 (13, 99)*	53 (38, 79)	71 (39, 85)
Triglicerydes (mg/dL)	80 (28, 191)	124 (66, 264)*	91 (28, 259)	105 (56, 218)
HDL-C (mg/dL)	47.3 (24.6, 79.5)	38.5 (21.7, 67.6)*	46 (32, 56)	37 (20, 60)
Glucose (mg/dL)	93 (76.6, 102)	97.5 (83.5, 115)	88.5 (76.6, 100.6)	92.6 (82.7, 101.2)
Others metabolic variables				
Insulin (μU/mL)	25 (8.3, 35.4)	33.4 (22.8, 64)*	14.9 (3.4, 43.9)	28.9 (16.9, 52.3)
HOMA-IR	4.8 (1.7, 7.0)	7.8 (5.2, 15.4)*	3.4 (0.68, 10.9)	6.6 (4.0, 11.4)
Adiponectin (μg/mL)	21.9 (7.7, 42.1)	20.2 (6.6, 57.3)	20.9 (11.6, 28.9)	15.3 (0.64, 33.0)
Adiponectin per Kg fat (μg/mL/kg)	1.7 (0.45, 14.3)	1.0 (0.27, 4.2)	1.9 (0.62, 4.2)	0.78 (0.06, 6.6)
Leptin ((μg/mL)	5.2 (1.8, 30.9)	21 (9.4, 40.7)*	6.2 (1.6, 25.1)	9.5 (3.6, 27.6)
Leptin per Kg fat (μg/mL/kg)	0.63 (0.13, 2.6)	1.1 (0.30, 4.4)*	0.39 (0.11, 4.8)	0.67 (0.13, 4.2)
Leptin to Adiponectin ratio	0.26 (0.05, 1.1)	1.2 (0.81, 3.5)*	0.34 (0.11, 1.2)	0.93 (0.11, 10.4)
Body Fat (%)	22 (11.2, 35.7)	39.8 (33.4, 49.3)*	23 (10.9, 43.4)	30.7 (18.7, 37.7)
VCAM (ng/mL)	801 (503, 2045)	818 (514, 1522)	617 (377, 1166)	727 (394, 1178)
ICAM (ng/mL)	404 (234, 753)	440 (200. 1124)	571 (184, 966)	411 (307, 1037)
MS				
Yes/No	0/18	7/14	0/9	3/5

Values are median (min, max); *Abbreviations*: *MS* Metabolic syndrome, *HOMA-IR* Homeostatic Model Assessment of Insulin Resistance, *VCAM* Vascular cell adhesion molecule, *ICAM* Intercellular adhesion molecule. Data compared Mann–Whitney *U*-test. Categorized Data were analyzed using Fisher’s Test. ^a^Three children of Chemotherapy group were excluded, because they were underweight; * *p* value < 0.05; **p value = 0.065

In the Table 5 are shown the analysis of the MS components and the aforementioned metabolic variables compared with biomarkers expressed in tertiles indicated that children who were classified in the highest tertile for BFP, leptin, and leptin/adiponectin ratio had a higher WC and higher levels of triglycerides, insulin, and HOMA index. Children in the highest tertile of the HOMA index had lower levels of adiponectin, and higher waist circumference, triglycerides, and leptin levels compared to children who were in the lowest tertile of the HOMA index.

**Table 5:** Analysis of metabolic variables with the components of metabolic syndrome at first assessment

Outcome variables	Fat percentage tertile (values)^a^		HOMA-IR tertile ^a^		Adiponectin tertile^a^		Leptin tertile^a^		Leptin adiponectin ratio tertile^a^	
	2 (24.2–35.0)	3 (35.1–46.7)	2 (3.6–5.5)	3 (5.6–13.9)	2 (15.3–25.4)	3 (25.5–44.3)	2 (4.1–13.4)	3 (13.5–30.8)	2 (0.3–0.7)	3 (0.8–2.9)
MS										
Waist circumference (cm)										
Coeficient	9.5	17.7	6.2	13.8	−1.9	−6.2	7.6	15.7	10.0	14.6
95% CI	3.4; 16.6	11.6; 23.7	−0.6; 13.1	6.9; 20.7	−10.1; 6.3	−14.4; 1.9	0.6; 14.6	8.7; 22.6	2.8; 17.1	7.4; 21.7
*P*	**0.033**	**0.000**	0.075	**<0.001**	0.641	0.133	**0.033**	**<0.001**	**0.007**	**<0.001**
Blood Pressure (mmHg)										
Systolic BP										
Coeficient	0.2	6.2	2.3	6.2	−1.4	−0.3	0.4375	5.4	3.1	7.6
95% CI	−6.4; 6.9	−0.5; 12.8	−4.4; 9.1	−0.4; 12.8	−8.3; 5.6	−7.4; 6.8	−6.4; 7.3	−1.4; 12.3	−3.6; 9.8	0.9; 14.3
*P*	0.944	0.068	0.488	0.066	0.693	0.940	0.898	0.117	0.353	**0.027**
Diastolic BP										
Coeficient	1.3	6.1	1.0	3.6	2.3	1.9	1.8	5.9	2.3	5.4
95% CI	−4.0; 7.1	0.3; 11.8	−5.0; 6.9	−2.3; 9.4	−4.0; 8.5	−4.4; 8.3	−4.4; 7.9	−0.3; 12.0	−3.9; 8.5	−0.8; 11.6
*P*	0.654	**0.040**	0.745	0.227	0.473	0.542	0.570	0.061	0.470	0.088
Glucose (mg/dL)										
Coeficient	4.9	0.1	4.9	10.6	−5.1	−0.6	4.2	2.3	1.3	2.9
95% CI	−0.8; 10.6	−5.6; 4.7	−0.1; 10.0	5.6; 15.7	−10.9; 0.8	−6.4; 5.2	−1.8; 10.1	−3.6; 8.3	−4.7; 7.3	−3.1; 8.9
*P*	0.089	0.983	0.055	**<0.001**	0.087	0.838	0.165	0.433	0.657	0.341
Triglycerides (mg/dL)										
Coeficient	28.4	52.3	36.6	38.8	3.7	−23.1	14.2	56.7	31.7	56.4
95% CI	−2.9; 59.8	20.9; 83.7	4.1; 69.0	6.3; 71.3	−30.8; 38.1	−57.5; 11.4	−16.7; 45.0	25.8; 87.6	0.5; 62.9	25.2; 87.7
*P*	0.075	**0.002**	**0.028**	**0.020**	0.832	0.184	0.360	**0.001**	**0.046**	**0.001**
HDL-C (mg/dL)										
Coeficient	−6.0	−3.0	−2.3	−8.2	−5.1	1.9	−1.1875	−4.9	−5.3	−9.4
95% CI	−14.5; 2.6	−11.6; 5.5	−10.6; 6.1	−16.6; 0.1	−13.2; 3.0	−6.2; 10.0	−9.4; 7.0	−13.1; 3.3	−13.2; 2.6	−17.3; −1.5
*P*	0.167	0.477	0.586	0.053	0.213	0.644	0.773	0.237	0.182	**0.021**
Adipokines										
Adiponectin (μg/mL)										
Coeficient	−4.6	4.3	0.3	−1.1			2.0	1.9	-	-
95% CI	−12.9; 3.8	−4.2; 12.8	−8.7; 9.3	−9.9; 7.8	-	-	−6.9; 10.8	−6.9; 10.7		
*P*	0.274	0.317	0.950	0.809			0.656	0.667		
Adiponectin (μg/mL/Kg)										
Coeficient	−0.7	−0.5	−0.5	−1.7			−0.5	−1.0	-	-
95% CI	−2.1; 0.6	−1.8; 0.9	−1.8; 0.7	−2.9; −0.5	-	-	−1.9; 0.9	−2.3; 0.4		
*P*	0.288	0.497	0.408	**0.009**			0.445	0.165		
Leptin (μg/mL)										
Coeficient	7.3	16.0	4.3	10.0	4.9	2.0	-		-	-
95% CI	4.6; 10.0	13.2; 18.8	−0.3; 9.0	5.4; 14.6	-0.3; 10.2	−3.2; 7.3				
*P*	**<0.001**	**<0.001**	0.069	**<0.001**	0.067	0.441				
Leptin (μg/mL/Kg)										
Coeficient	0.6	1.0	0.7	0.3	0.7	0.1	-	-	-	-
95% CI	0.1; 1.2	0.4; 1.5	0.1; 1.3	−0.3; 0.9	0.1; 1.2	-0.4; 0.7				
*P*	**0.048**	**0.001**	**0.037**	0.341	**0.032**	0.699				
Insulin resistance										
Fasting insulin (mμ/mL)										
Coeficient	13.3	10.6	-	-	−2.2	−2.3	10.6	15.0	11.2	13.1
95% CI	7.0; 19.6	4.3; 16.9			−10.1; 5.6	−10.1; 5.5	4.2; 17.0	8.6; 21.4	4.6; 17.9	6.4; 19.8
*P*	**<0.001**	**0.001**			0.569	0.559	**0.002**	**<0.001**	**0.001**	**<0.001**
HOMA-IR										
Coeficient	3.2	2.3	-	-	−0.8	−0.6	2.6	3.4	2.6	3.1
95% CI	1.6; 4.9	0.7; 3.9			−2.8; 1.1	−2.5; 1.3	0.9; 4.3	1.7; 5.1	0.9; 4.3	1.3; 4.8
*P*	**<0.001**	**0.007**			0.391	0.537	**0.003**	**<0.001**	**0.004**	**0.001**

Values obtained through robust linear regression analysis, adjusted for gender, age, treatment (only chemotherapy or chemotherapy plus radiotherapy), and diagnoses (acute lymphoblastic leukemia or lymphoma). ^a^The referent was tertile 1. *Abbreviations*: *MS* Metabolic syndrome

At 6 months, utilizing the tertile without changes as a reference (Table 6), children who reduced their BFP also reduced their WC and insulin concentration. However, the children who increased HOMA index values had a 2.5 cm greater WC, 5.1 mg/dL higher glucose, and 30 mg/dL higher triglycerides. In contrast to these findings, when children reduced their leptin concentrations, their glucose levels decreased. On the contrary, when they reduced their adiponectin values, their HDL-C levels also decreased. Similarly, when the leptin/adiponectin ratio tertile decreased, decreases in the levels of glucose, triglycerides, fasting insulin and HOMA index were also observed.

**Table 6:** Analysis of metabolic variables with the components of metabolic syndrome at 6-months

Outcome variables	Fat percentage Change tertiles (values)		HOMA-IR Change tertiles (values)		Adiponectin Change tertile (values)		Leptin Change tertile (values)		Leptin adiponectin ratio tertile (values)	
	Decreased (−5.9 – −0.7)	Increased (1.3–5.6)	Decreased (−7.2–0.2)	Increased (1.8–7.3)	Decreased (−55.6 – −3.2)	Increased (2.1–21.9)	Decreased (−13.4 – −0.4)	Increased (2.7–17.5)	Decreased (−1.1 – −0.01)	Increased (0.1–1.5)
MS										
Waist circumference (cm)										
Coeficient	−3.8	0.6	−0.1	2.5	−0.3	0.6	−0.1	2.7	−1.5	2.5
95% CI	−6.2;-1.4	−1.7;2.8	−2.4; 2.3	0.1; 4.9	−2.9; 2.4	−2.2; 3.3	−2.6; 2.5	0.1; 5.3	−3.9; 0.9	0.1; 5.0
*P*	**0.003**	0.623	0.950	**0.041**	0.850	0.668	0.963	**0.041**	0.218	**0.042**
Blood Pressure (mmHg)										
Systolic BP										
Coeficient	0.1	−6.8	5.1	6.7	−3.5	−4.0	−3.5	−1.7	−7.3	−5.4
95% CI	−9.2; 9.5	−15.5;1.9	−2.8; 13.1	−1.3; 14.6	−12.3; 5.3	−12.8; 4.8	−12.5; 5.6	−10.9; 7.5	−16.1; 1.6	−14.3; 3.6
*P*	0.975	0.123	0.202	0.098	0.425	0.361	0.447	0.709	0.104	0.235
Diastolic BP										
Coeficient	−5.6	−7.7	6.1	3.6	−2.9	2.2	−5.1	−0.2	−3.4	−3.5
95% CI	−13.9; 2.6	−15.3;-0.0	−1.1; 13.3	−3.5; 10.8	−10.3; 4.5	−5.1; 9.6	−12.6; 2.4	−7.9; 7.4	−11.1; 4.2	−11.3; 4.2
*P*	0.175	0.049	0.093	0.312	0.433	0.542	0.180	0.955	0.375	0.366
Glucose										
Coeficient	0.3	1.9	−5.5	5.1	−2.0	−1.7	−6.9	1.7	−5.9	1.5
95% CI	−6.4; 6.9	−4.4; 8.1	−10.4; −0.7	0.2; 10.0	−7.9; 4.0	−7.8; 4.4	−12.3; -1.5	−3.7;7.2	−11.5; 0.4	−4.1; 7.2
*P*	0.936	0.549	**0.027**	**0.043**	0.509	0.568	**0.013**	0.529	**0.037**	0.590
Triglycerides										
Coeficient	−3.8	−5.4	−3.6	30.0	9.4	1.7	−8.9	2.9	−40.7	−10.8
95% CI	−37.7; 30.2	−37.6; 26.8	−31.5; 24.3	1.7; 58.3	−22.2; 41.0	−30.5; 33.8	−41.1; 23.2	−29.8; 35.5	−70.4; −11.1	−40.9; 19.4
*P*	0.823	0.738	0.797	**0.038**	0.551	0.918	0.579	0.861	**0.008**	0.476
HDL-C										
Coeficient	0.6	−1.8	2.4	3.6	−8.3	−0.5	1.0	3.0	2.6	0.3
95% CI	−7.1; 8.4	−9.0; 5.4	−4.2; 9.0	−3.2; 10.4	−14.5; −2.1	−6.9; 5.9	−6.0; 8.0	−4.2; 10.2	−4.4; 9.6	−6.9; 7.6
*P*	0.867	0.620	0.469	0.288	**0.010**	0.886	0.770	0.401	0.453	0.926
Adipokines										
Adiponectin										
Coeficient	0.1	−3.9	5.8	2.5	-	-	−1.2	−2.4	-	-
95% CI	−4.0; 4.8	−9.0; 1.3	1.3; 10.3	−2.3; 7.3			−6.1; 3.8	−7.4; 2.6		
*P*	0.980	0.138	**0.013**	0.298			0.639	0.332		
Leptin										
Coeficient	0.3	1.4	−1.8	1.1	2.8	3.5	-	-	-	-
95% CI	−4.3; 4.9	−2.9; 5.6	−5.5; 1.9	−2.8; 5.0	−0.8; 6.5	−0.2; 7.1				
*P*	0.895	0.511	0.331	0.569	0.130	0.063				
Insulin resistance										
Fasting insulin (mμ/mL)										
Coeficient	−6.9	0.1	-	-	−1.1	−6.3	−4.1	2.5	−7.6	2.4
95% CI	−12.7; -1.1	−5.8; 6.0			−7.8; 5.7	−13.2; 0.6	−11.1; 2.9	−4.6; 9.6	−14.1; −1.1	−4.3; 9.0
*P*	**0.020**	0.978			0.753	0.072	0.241	0.480	**0.023**	0.476
HOMA-IR										
Coeficient	1.8	1.7	-	-	−0.4	−1.7	−1.1	0.9	−2.2	0.8
95% CI	0.1; 3.4	0.1; 3.4			−2.2; 1.5	−3.6; 0.2	−3.1; 0.8	−1.0; 2.9	−3.9; -0.4	−1.0; 2.6
*P*	**0.033**	**0.043**			0.708	0.083	0.237	0.345	**0.017**	0.387

Values obtained through robust linear regression analysis, adjusted for gender, age, treatment (only chemotherapy or chemotherapy plus radiotherapy), and and diagnoses (acute lymphoblastic leukemia or lymphoma). Regression coefficients are change in outcome variable per tertile change in fat percentage, HOMA-IR Adiponectin, leptin, and Leptin adiponectin ratio. The referent was stable tertile

Table 7 shows that the odds of developing MS at the first assessment were higher in those with a BFP (OR 5.1, 95% CI 1.9 to 22.0), leptin level (OR 4.8, 95% CI 1.4 to 17.2) and leptin/adiponectin ratio (OR 5.2 95% CI 1.2 to 22.6) in the highest tertile, whereas the lowest tertile of adiponectin was associated with a protective but not significant effect. Similar findings were also observed after 6 months. The HOMA index only was a predictive factor of developing MS at 6 months.

**Table 7:** Odds ratio of developing metabolic syndrome according to different metabolic variables

Variable	First assessment			After 6 months		
	OR^a^	95% IC	*P*	OR^a^	95% IC	*p*
Body fat percentage	5.1	1.9; 22.0	**0.028**	9.0	2.0; 41.1	**0.004**
HOMA-IR index	2.3	0.2; 1.4	0.137	5.5	1.6; 19.3	**0.007**
Leptin	4.8	1.4; 17.2	**0.015**	5.7	1.6; 20.2	**0.007**
Adiponectin	0.5	0.2; 1.5	0.189	0.9	0.4; 2.0	**0.728**
Leptin to adiponectin ratio	5.2	1.2; 22.6	**0.027**	9.4	2.0; 43.8	**0.004**

^a^OR obtained through logistic regression, by tertil change and adjusted for gender, age, treatment (only chemotherapy or chemotherapy plus radiotherapy), and diagnoses (acute lymphoblastic leukemia or lymphoma)

## Discussion

Our study showed that during early surveillance, the adiposity, HOMA-IR index, concentration of leptin, and leptin/adiponectin ratio are strongly associated with MS development in children surviving lymphoma and ALL. In contrast, the adiponectin concentration seems to have a protective effect on the development of this syndrome.

In our sample of child cancer survivors, the estimated frequencies of MS tended to be greater than those observed by Kourti (5.8%) [22], Trimis [23] (11.2%), Chow [24] (10.8%) and Aldhafiri [25] (7.1, 5.4%) and similar to those reported by Reisi [26] (20%). This variation in the frequency of MS is principally due to the lack of consistent criteria and cut-off points in its definition. However, our results are similar to several studies conducted in Mexico in an open pediatric population that used the same definition. For example, the Juarez et al. study [27], reports a prevalence of 20% in children and obese adolescents with ages from 11 to 13 years, whereas Klunder et al. [14] reported a prevalence of 13% in obese children 6–12 years.

In our Country, there are no statistics regarding the number of survivors of childhood cancer reaching adulthood. However, within the Hematology and Oncology Services at our hospital, we observed a gradual increase in this group of patients. Mexico is a country with the highest prevalence of overweight and obesity in the world. In this context, MS is considered to be a complex clinical condition that is associated with early obesity and other metabolic risk factors associated with the start of its development during childhood. In various studies, an increase in the frequency of obesity in survivors of childhood cancer has been observed [3, 28]. We also found that the percentages of leukemia and lymphoma survivors presented combined prevalence rates of overweight and obesity that were slightly greater (38 and 42%) than those reported by the National Health Survey (ENSANUT 2012) [29] in a Mexican pediatric population (35%). This finding is relevant given that central obesity can cause insulin resistance, a metabolic alteration that is found in the majority of individuals with MS and which increases the risk of type 2 diabetes and cardiovascular disease [30, 31]. In this study, approximately 80% of the surviving youths presented high levels of insulin and resistance to insulin. In these children, hyperinsulinemia compensates for insulin resistance and maintains the homeostasis of insulin. However, even without alterations in the metabolism of glucose, the risk of presenting with additional health problems exists, such as early atherosclerosis, hypertension, and so on.

Currently, given the increase in complications associated with MS, various molecules have been proposed to predict the risk of this syndrome in both general population and pediatric cancer survivor patients. The main observations of this study were that childhood cancer survivors in the highest tertile of the leptin/adiponectin ratio, BFP, and HOMA index presented with lower levels of adiponectin per/kg/fat, a greater waist circumference, high concentrations of leptin and triglycerides, a higher systolic blood pressure and lower HDL-C concentrations. In this study, BFP and the leptin-to-adiponectin ratio were strong predictors of MS, with odds ratios of 9 and 9.4, respectively. For this reason, the measurement of these metabolic variables in early surveillance screenings can be useful to identify individuals who are susceptible to metabolic risk. In the leptin/adiponectin relationship, this measurement can be considered to be an indicator of sensitivity to insulin in youth who have survived childhood cancer. However, it should be noted that adiponectin, an adipocytokine with antiatherogenic, antidiabetogenic, and anti-inflammatory functions that is known as a predictor of MS in children [7, 14, 32] did not show this association. This observation can most likely be explained by the high number of participants who presented with IR, as previous studies revealed that individuals with IR present lower serum levels of adiponectin, independent of sex, age and ethnicity [33, 34]. Similarly, the change in these indicators demonstrated a direct effect on the metabolic profiles of children. In contrast, an increase in the concentration of adiponectin at 6 months showed a trend toward reduced concentrations of insulin and HOMA index, without reaching statistical significance. In child cancer survivors, few studies have evaluated the role of these adipokines, in addition to levels of insulin, adiposity and the presence of MS. Tonorezos et al. [35], in a cross-sectional study of 116 adult survivors of childhood leukemia (median age, 23 years), reported that survivors presented high levels of leptin associated with body fat and insulin resistance. In a more recent study that is similar to ours, Kojima et al. [36] reported that low levels of total adiponectin were associated with a greater number of patients with hypertriglyceridemia and hypertension.

Based on these results, we should discuss the strengths and limitations of our work. Given the nature of the study, survival bias could not be avoided, as only patients who were alive at the time of the study was conducted could be evaluated. This bias is important because in developing countries, such as Mexico, the survival rate of children with ALL at 5 years is less than that reported in developed countries (73 vs 90%) [37]. Therefore, our findings could not be extrapolated to developed countries. However, in developing countries with survival rates similar to ours, the results could have external validity. It was also difficult to determine whether the percentage of obesity found in these children is primarily related to the treatment received (chemotherapy, radiation therapy or both), as the nutritional state of the metabolic variables at the time of diagnosis and during treatment is not known. However, currently, various studies conducted in this population have indicated an increase in the incidence of obesity and resistance to insulin related to treatment in ALL and lymphoma survivors during therapy [38]. The development of these metabolic alterations has been related to a deficiency in the secretion of growth hormone, not only in those patients who received brain radiation but also in those who have received chemotherapy or stem cell transplants [6]. In this study, survivors of childhood ALL and lymphoma are at increased risk of short stature, the risk is highest for those treated with radiotherapy. Another possible explanation for the increased risk of developing components of MS in this population is related to corticosteroid therapy, as it is widely accepted that children who receive steroids gain weight [6]. An additional limitation was that pubertal stage was determined on the basis of the children’s self-assessment using Tanner’s standard photographs for pubic hair and genital development, which is a more subjective measure, as it depends on the patient’s self-assessment. Validation studies showed that self-assessment is inferior to clinical assessment [39]. In addition, in this study, we have not analyzed what kinds of food they were eating and their physical activity. On the other hand, the presence of MS increases cardiovascular risk and vascular brain disease as a consequence of premature changes in the arterial wall, including endothelial cell damage [40]. In this sample of child cancer survivors, endothelial function (in terms of VCAM and ICAM) was not different between those who received only chemotherapy and those who received radiation therapy and between those who were diagnosed with MS and those who did not develop MS. To validate that the findings were not due to chance, a second measurement at 6 months was conducted. This second measurement confirmed that most of the findings observed in the first measurement were stable at 6 months. Variables such as BFP were obtained using the DXA technique, which is considered to be reliable and precise in children. With regard to the metabolic and biochemical variables, standard techniques were employed to quantify their concentration in a specialized research laboratory. It was determined that a greater number of metabolic variables were reported than in other studies.

## Conclusions

Biomarkers such as HOMA-IR, leptin and leptin/adiponectin are associated with each of the components of the MS and with a heightened risk of suffering MS among children survivors of cancer. Given the close relationship between MS with risk of developing type 2 diabetes and cardiovascular diseases, it is imperative to implement prevention measures in this population and especially in developing countries where these pathologies have become the leading cause of death.

## Abbreviations

ALL: Acute linfoblastic leukemia
BFP: Body fat percentage
BMI: Body mass index
CDC: Center for disease control and prevention
DXA: Dual energy X ray absorptiometry
HDL-C: High density lipoprotein cholesterol
HOMA-IR: Homeostatic model assessment insulin resistance
iCAM: Intracellular adhesion molecule
IR: Insulin resistance
MS: Metabolic syndrome
OR: Odds ratio
vCAM: Vascular cell adhesion molecule
WC: Waist circumference
